# Supplementary material for: Exploring the Cognitive Capacities of Japanese Macaques in a Cooperation Game
Source: Animals (Basel). 2021 May 21;11(6):1497. doi: 10.3390/ani11061497 (PMC8224363; doi:10.3390/ani11061497)
Supplement: Supplementary file 1 [file animals-11-01497-s001.zip › animals-1204814-supplementary/Sigmundson, Stribos, et al. Experiment 2 Script.pdf]

R-Script analysis experiment 2

```
library(lme4)

install.packages
library(lmerTest)
library(car)
library(MASS)
library(fitdistrplus)
library(survival)
library(fitdistrplus)
library(robustlmm)
library(arm)
library(MASS)
library(Matrix)
library(pscl)
library(R2admb)
library(glmmADMB)
library(MuMIn)
library(pbkrtest)
library(emmeans)
library(irr)
library(ggplot2)
```

#First hurdle Binomial on Stress 1st Min#

```
StrBinMin1 <- glmer(StrBinMin1~Outcome+(1|Focal/Partner/Observer/Reward.type), data= xdata,
family = "binomial")

summary(StrBinMin1)

emmeans(StrBinMin1, list(pairwise ~ Outcome))

StrBinMin1Null <- glmer(StrBinMin1~(1|Focal/Partner/Observer/Reward.type), data= xdata, family =
"binomial")

anova(StrBinMin1,StrBinMin1Null)
```

#Second hurdle Linear on Stress>0 1st Min#

```
StressMin1 <- glmer.nb(StressMin1~Outcome+(1|Focal/Partner/Observer/Reward.type),

        data=subset(xdata,StressMin1>0))

summary(StressMin1)

StressMin1Null <- glmer.nb(StressMin1~(1|Focal/Partner),

        data=subset(xdata,StressMin1>0))

anova(StressMin1,StressMin1Null)
```

#First hurdle Binomial on Aggr 1st Min#

```
AggBinMin1 <- glmer(AggBinMin1~Outcome+(1|Focal/Partner/Observer/Reward.type), data= xdata,
family = "binomial")

summary(AggBinMin1)

emmeans(AggBinMin1, list(pairwise ~ Outcome))

AggBinMin1Null <- glmer(AggBinMin1~(1|Focal/Partner/Observer/Reward.type), data= xdata, family
= "binomial")

anova(AggBinMin1,AggBinMin1Null)
```

#Second hurdle Linear on Aggr>0 1st Min#

```
AggMin1 <- glmer.nb(AggMin1~Outcome+(1|Focal/Partner/Observer/Reward.type),

            data=subset(xdata,AggMin1>0))

summary(AggMin1)

AggMin1Null <- glmer.nb(AggMin1~(1|Focal/Partner),

              data=subset(xdata,AggMin1>0))

anova(AggMin1,AggMin1Null)
```

#First hurdle Binomial on Stress over 3min#

```
StressBin <- glmer(StressBin~Outcome+(1|Focal/Partner/Observer/Reward.type), data= xdata, family
= "binomial")

summary(StressBin)

emmeans(StressBin, list(pairwise ~ Outcome))

StressBinNull <- glmer(StressBin~(1|Focal/Partner/Observer/Reward.type), data= xdata, family =
"binomial")

anova(StressBin,StressBinNull)

anova(StressBin)
```

#Second hurdle Linear on Stress>0 over 3min#

```
Stress3Min <- glmer.nb(Stress3Min~Outcome+(1|Focal/Partner/Observer/Reward.type),

            data=subset(xdata,Stress3Min>0))

summary(Stress3Min)

Stress3MinNull <- glmer.nb(Stress3Min~(1|Focal/Partner/Observer/Reward.type),

              data=subset(xdata,Stress3Min>0))

anova(Stress3Min,Stress3MinNull)
```

#First hurdle Binomial on Aggr#

```
AggBin <- glmer(AggBin~Outcome+(1|Focal/Partner/Observer/Reward.type), data= xdata, family = "binomial")

summary(AggBin)

emmeans(AggBin, list(pairwise ~ Outcome))

anova(AggBin)

AggBinNull <- glmer(AggBin~(1|Focal/Partner/Observer/Reward.type), data= xdata, family = "binomial")

anova(AggBin,AggBinNull)
```

#Second hurdle Linear on Aggr>0 3min#

```
Agg3Min <- glmer.nb(Agg3Min~Outcome+(1|Focal/Partner/Observer/Reward.type),

          data=subset(xdata,Agg3Min>0))

summary(Agg3Min)

Agg3MinNull <- glmer.nb(Agg3Min~(1|Focal/Partner/Observer/Reward.type),

            data=subset(xdata,Agg3Min>0))

anova(Agg3Min,Agg3MinNull)
```

#Effect of cumulative value of trial on stress#

```
StressCondCumx<-glmer(`Stress bnm overall`~Con.worth+cu.worth+(1|Focal),data = cu_avx, family = binomial(link = logit))

summary(StressCondCumx)

StressCondx<-glmer(`Stress bnm overall`~Con.worth+(1|Focal),data = cu_avx, family = binomial(link = logit))

summary(StressCondx)

anova(StressCondx,StressCondCumx)

StressCumx<-glmer(`Stress bnm overall`~cu.worth+(1|Focal),data = cu_avx, family = binomial(link = logit))

summary(StressCumx)

StressCumCondx<-glmer(`Stress bnm overall`~cu.worth+Con.worth+(1|Focal),data = cu_avx, family = binomial(link = logit))

summary(StressCumCondx)

anova(StressCondx,StressCumx)
```

Stressnullx<-glmer(`Stress bnm overall`~(1|Focal),data = cu_avx, family = binomial(link = logit))

anova(StressCondx, Stressnullx, StressCondCumx, StressCumx)

#Effect of cumulative value of trial on aggression#

AggCondCumx<-glmer(`Aggression bnm overall`~Con.worth+cu.worth+(1|Focal),data = cu_avx, family = binomial(link = logit))

summary(AggCondCumx)

AggCondx= glmer(`Aggression bnm overall`~Con.worth+(1|Focal),data = cu_avx, family = binomial(link = logit))

summary(AggCondx)

anova(AggCondx,AggCondCumx)

AggCumx= glmer(`Aggression bnm overall`~cu.worth+(1|Focal),data = cu_avx, family = binomial(link = logit))

summary(AggCumx)

anova(AggCumx,AggCondx)

Aggnullx= glmer(`Aggression bnm overall`~(1|Focal),data = cu_avx, family = binomial(link = logit))

anova(AggCondx,Aggnullx, AggCondCumx, AggCumx)

#Effect of looking in the box before cooperation that resulted in outcome B#
#first make a sub-dataset of only outcome B#

#on Stress
StressBinLookB <- glmer(StressBin~Lookbox+(1|Focal/Partner/Observer/Reward.type), data= xdata, family = "binomial")

summary(StressBinLookB)

StressBinNullB <- glmer(StressBin~(1|Focal/Partner/Observer/Reward.type), data= xdata, family = "binomial")

anova(StressBinLook,StressBinNull)

#on Aggression
AggBinLookB <- glmer(AggBin~Lookbox+(1|Focal/Partner/Observer/Reward.type), data= xdata, family = "binomial")

summary(AggBinLookB)

anova(StressBinLook,StressBin)

AggBinNullB <- glmer(AggBin~(1|Focal/Partner/Observer/Reward.type), data= xdata, family = "binomial")

anova(AggBinLook,AggBinNull)
