# Supplementary material for: Exploring the Cognitive Capacities of Japanese Macaques in a Cooperation Game
Source: Animals (Basel). 2021 May 21;11(6):1497. doi: 10.3390/ani11061497 (PMC8224363; doi:10.3390/ani11061497)
Supplement: Supplementary file 1 [file animals-11-01497-s001.zip › animals-1204814-supplementary/Sigmundson, Stribos, et al. Supplementary materials.pdf]

Supplementary materials for Sigmundson, Stribos, et al. (2021). Exploring the cognitive capacities of Japanese macaques in a cooperation game. ***Animals*****.**

Table S1. Summary of Results by Dyad

Dyad	Partner Relationship	Total Successes
James + Ingrid	Son-Mother	228
James + Kate	Nephew-Aunt	175
James + Pippi	Non-kin	145
James + Finn	Non-kin	64
Pippi + Alina	Daughter-Mother	54
Finn + Herta	Non-kin	10
James + Montana	Non-kin	10
James + Alina	Non-kin	4
Finn + Krato	Non-kin	3
James + Herta	Non-kin	3
James + Julia	Non-kin	2
James + Krato	Non-kin	2
James + Sandra	Non-kin	1
Kate + Sandra	Non-kin	1
Ingrid + Sandra	Non-kin	1
Pippi + Herta	Non-kin	1
Pippi + Kate	Non-kin	1
Pippi + Finn	Non-kin	1
James + Kurtney	Non-kin	1
Pippi + Ingrid	Non-kin	1

Number of successes during Experiment 1, broken down by dyad. Kin relationship of the dyad is shown in the middle column. Only dyads with at least one successful cooperation are shown. All other possible dyadic combinations can be assumed to have zero successful attempts.

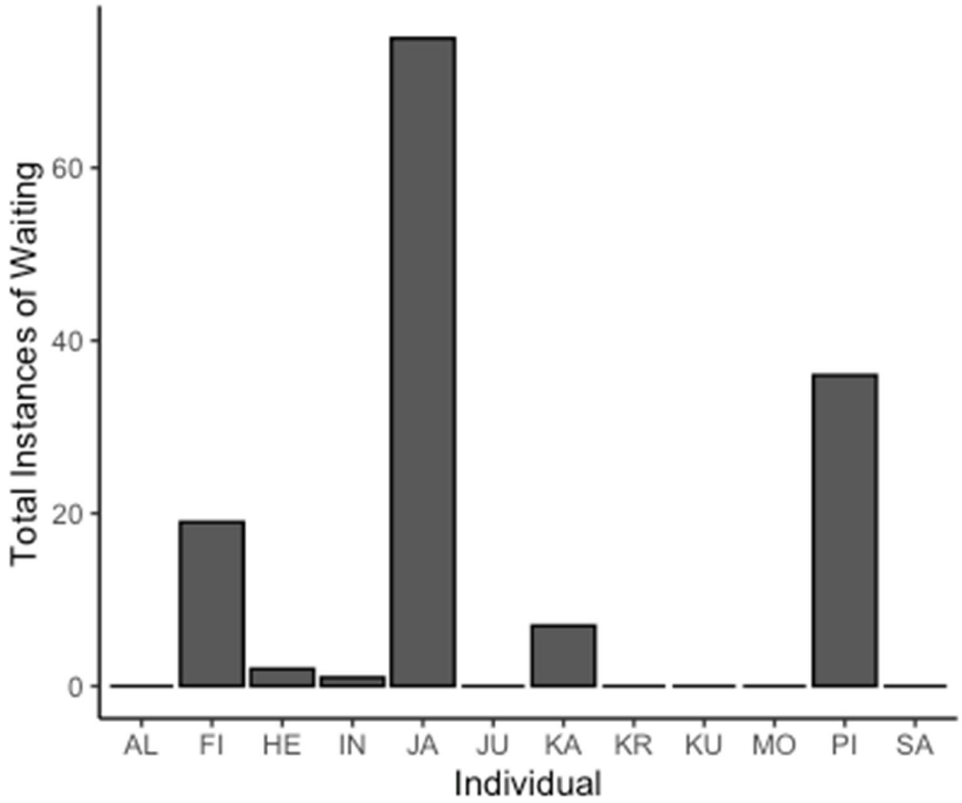

Figure S1. Bar graph displaying total instances of waiting during Experiment 1 broken down by individual. Individual labels are composed of the first two letters of the corresponding monkey's name.

Table S2. Definitions of Behavioral Variables Recorded during Experiment 2

**Behavior**	**Description**
Refusal to cooperate	Focal has cooperated before in this session with an individual that is currently in the hut waiting close to one of the strings. Focal shows no interest in acting as cooperation partner again with that individual for at least 10 seconds
Look in box	Focal inspects the box for food rewards prior to cooperation. This involves climbing up on the apparatus and directing gaze to the plexiglass windows of the box
Stress-related behaviors	
Scratch	Focal uses finger, hand or foot to rake across own skin
Body shake	Focal rapidly turns the whole body in at least two different directions alongside the axis of its spine
Yawn	Focal opens mouth wide and inhales intensely, which can be seen by the expansion of the chest
Aggressive behaviors	
Bite	Focal places its teeth on the body of another individual and closes its jaws
Chase	Focal runs after another individual for at least 3 meters
Grunt	Focal makes a low pitch rumbling vocalization with focus toward another individual
Hit	Focal slaps another individual with its hand
Lunge	Focal jumps a maximum of two body lengths forward toward another individual
Physical Attack	Focal commits at least two consecutive acts of aggression against another individual (may include grab, hit, and/or bite)
Stare	Focal looks intensively at another individual with a straight back and raised eyebrows
Threat	Focal moves its head with an O-shaped mouth, retracted lips, and raised eyebrows toward another individual
